# Molecular identification of streptomycin monoresistant Mycobacterium tuberculosis related to multidrug-resistant W strain.

**DOI:** 10.3201/eid0705.010512

**Published:** 2001

**Authors:** P Bifani, B Mathema, M Campo, S Moghazeh, B Nivin, E Shashkina, J Driscoll, S S Munsiff, R Frothingham, B N Kreiswirth

**Affiliations:** Public Health Institute Tuberculosis Center, New York, NY 10016, USA.

## Abstract

A distinct branch of the Mycobacterium tuberculosis W phylogenetic lineage (W14 group) has been identified and characterized by various genotyping techniques. The W14 group comprises three strain variants: W14, W23, and W26, which accounted for 26 clinical isolates from the New York City metropolitan area. The W14 group shares a unique IS6110 hybridizing banding motif as well as distinct polymorphic GC-rich repetitive sequence and variable number tandem repeat patterns. All W14 group members have high levels of streptomycin resistance. When the streptomycin resistance rpsL target gene was sequenced, all members of this strain family had an identical mutation in codon 43. Patients infected with the W14 group were primarily of non- Hispanic black origin (77%); all were US-born. Including HIV positivity, 84% of the patients had at least one known risk factor for tuberculosis.


### Molecular Identification of Streptomycin Monoresistant *Mycobacterium tuberculosis* Related to Multidrug-Resistant W Strain

**Pablo Bifani,*† Barun Mathema,* Martha Campo,* Soraya Moghazeh,* Beth Nivin,‡ Elena Shashkina,* Jeffrey Driscoll,§ Sonal S. Munsiff,‡ Richard Frothingham,¶ and Barry N. Kreiswirth***

*Public Health Research Institute Tuberculosis Center, New York, NY, USA; †New York University School of Medicine, New York, New York, USA; ‡New York City Department of Health, New York, New York, USA; §New York State Department of Health, Albany, New York, USA; ¶Durham Veterans Affairs Medical Center, Durham, North Carolina

A distinct branch of the *Mycobacterium tuberculosis* W phylogenetic lineage (W14 group) has been identified and characterized by various genotyping techniques. The W14 group comprises three strain variants: W14, W23, and W26, which accounted for 26 clinical isolates from the New York City metropolitan area. The W14 group shares a unique IS*6110* hybridizing banding motif as well as distinct polymorphic GC-rich repetitive sequence and variable number tandem repeat patterns. All W14 group members have high levels of streptomycin resistance. When the streptomycin resistance *rps*L target gene was sequenced, all members of this strain family had an identical mutation in codon 43. Patients infected with the W14 group were primarily of non-Hispanic black origin (77%); all were US-born. Including HIV positivity, 84% of the patients had at least one known risk factor for tuberculosis.

With the advent of molecular techniques, tuberculosis (TB) investigators have a powerful tool to further the understanding of the transmission and phylogenetic properties of *Mycobacterium tuberculosis*. Molecular techniques have been used to discriminate exogenous versus endogenous disease (1-3), investigate suspected outbreaks (2,4-6) and cases of laboratory cross-contamination (7-9), study transmission within a defined geographic setting (10,11), and demonstrate the occurrence of exogenous superinfection in immunocompetent and immunocompromised patients (12,13).

Genotyping has facilitated identification and characterization of the W strain, a multidrug-resistant (MDR) clone associated primarily with nosocomial transmission in hospitals and detention facilities in New York City (NYC) in the early 1990s (14-17). In such studies, molecular markers were used to confirm and characterize the W strain outbreak and to elucidate a plausible evolutionary scenario for the sequential acquisition of multidrug resistance (14,17,18). In a recent study, when molecular techniques were applied in a population-based setting, genotyping identified a drug-susceptible group of isolates (W4) that represents a distinct branch of the W phylogenetic lineage. Members of this W4 group define a previously unidentified cluster of cases in a community in northern New Jersey; the cluster likely resulted from both historical and recent transmission (19). Although the W-MDR strain from NYC is clearly distinct from the drug-susceptible W4 group, both groups of isolates evolved from a common ancestor (20).

Strains that define the W family have several distinguishing genotypes in common: 1) they belong to principal genetic group 1 (17,21); 2) they have similar spoligotype patterns, characterized by a deletion of spacers 1-34 and the corresponding repeat in the direct repeats (DR) region (19, 22-24), an alteration that defines spoligopattern S00034 and closely related spoligotypes; 3) they contain a unique insertion in the origin of replication (19,20) and in the NTF locus (20,25). Strains grouped in the W or Beijing family are prevalent in China, Southeast Asia (26,27), Russia, and other former Soviet regions (unpub. data) and have recently been reported in South Africa (23).

By multiple genetic techniques, we investigated a cluster of NYC *M. tuberculosis* isolates (W14 group) that are resistant to at least streptomycin and share identical or closely related DNA fingerprint patterns. Results from multiple typing methods were used to show the relatedness of the three IS*6110* fingerprint patterns, W14, W23, and W26 and their evolution.

### Materials and Methods

### Mycobacterial Isolates and Patients

A total of 26 isolates from 26 patients were identified and grouped based on their IS*6110* fingerprint patterns. The IS*6110* fingerprint archive at the Public Health Research Institute Tuberculosis Center (PHRI TB Center) includes fingerprint patterns from >13,000 *M. tuberculosis* clinical

*Address for correspondence: Barry N. Kreiswirth, Public Health Research Institute Tuberculosis Center, New York, New York 10016; USA; fax: 212-578-0853; e-mail: barry@phri.org specimens characterized since 1992. More than 80% of the isolates were cultured from NYC and New Jersey cases; remaining samples are from seven other states and international sources.

The W14 group isolates are part of a NYC convenience sample, which represents 44% (n=6,655) of the total number of culture- positive TB cases reported in 1992-1999 (~15,000). Isolates were collected for numerous outbreak, surveillance, and research studies (14,17,28-31). Basic clinical, demographic, and routine contact-tracing information was obtained for 22 of the 26 patients; data for 21 cases were obtained from the NYC Tuberculosis Control Program surveillance database; the one exception was from the New Jersey Department of Health and Senior Services. Isolates originated from 16 institutions, including NYC hospitals and correctional facilities. Laboratory cross-contamination was ruled out since none of the clinical specimens were cultured or processed during the same time period, and all patients had well-documented TB.

### IS*6110* DNA Fingerprinting and Pattern Interpretation

*M. tuberculosis* isolates were cultured on Lowenstein-Jensen slants and grown at 37°C for 3 to 5 weeks. IS*6110* DNA fingerprint analyses were performed according to a standard method using both the 5' and 3' fragments of the IS*6110* genetic element (32). Hybridization patterns were compared on a Sun Sparc5 Workstation by using the BioImage Whole Band Analyzer software version 3.4 (Genomic Solutions, Ann Arbor, MI). The Jaccard matching method and an unweighted pair group method that used arithmetic averages and average linkage clustering identified related patterns, in accordance with the protocol of the National Tuberculosis Genotyping and Surveillance Network, Centers for Disease Control and Prevention (CDC). Nomenclature of the DNA fingerprint patterns was as follows: Isolates with identical banding patterns were assigned the same arbitrary letter code (e.g., W, J, AF). IS*6110* patterns that resembled but were not identical to one of these patterns were denoted by addition of a number (e.g., W14, W23).

### Other Southern Blot Hybridization Probes: Polymorphic GC-Rich Repetitive Sequence and Direct Repeat

Chromosomal DNA was restricted with *Alu* I and hybridized with the polymorphic GC-rich repetitive sequence (PGRS) probe (GenBank accession number M95490) (33). Direct-repeat restriction fragment-length polymorphism (RFLP) primers DRa and DRb were used to generate a DR probe with H37Rv (strain ATCC 35177) as a template. The DR amplicon was used as a probe to re-hybridize both membranes (*Pvu* II and *Alu* I) previously generated for IS*6110* and PGRS genotyping, respectively (34).

### Spacer Oligonucleotide Genotyping (Spoligotyping)

Spoligotyping was performed according to the protocol described by Kamerbeek et al. (34). The spoligotype of the 26 samples belonging to the W14 group was compared against a spoligotype database maintained by the Wadsworth Center, New York State Department of Health, comprising >2,500 clinical specimens.

### Variable Number of Tandem Repeats

Tandem repeat loci ETR-A to ETR-E were amplified by polymerase chain reaction (PCR) and analyzed by gel electrophoresis to generate variable number of tandem-repeat (VNTR) allele profiles, as described by Frothingham and Meeker-O'Connell (35). Each digit of the allele profile represents the number of tandem-repeat copies at a particular locus. The patterns were compared against the PHRI TB Center VNTR database (~500 isolates) and profiles at the Durham Veterans Affairs Medical Centers database (745 isolates, 85 in the W phylogenetic lineage) (19).

### Streptomycin/Isoniazid/Rifampin/Ethambutol and Ethionamide Susceptibility Testing

Primary drug resistance to streptomycin/isoniazid (INH)/rifampin/ethambutol (SIRE) was determined by using TB susceptibility Quad Plate I and II (Remel No. 3501). *M. tuberculosis* cultures were determined to be resistant to antimicrobial agents at concentrations $\geq$1 µg/mL for INH and rifampin, 7.5 µg/mL for ethambutol, and 10 µg/mL for streptomycin. The MIC for streptomycin was determined for representative samples according to standard methodology by using the agar diffusion assay (36). Isolates were subcultured onto 7H10/ADC medium (Difco, Detroit, MI) containing 2 to 500 µg/mL of streptomycin. The NYC Bureau of Laboratories performed pyrazinamide (PZA) testing (37).

### DNA Sequencing Streptomycin (*rps*L) and INH (kat G) Resistance Genes

Analyses of the *rpsL* and *katG* genes were performed on a MicroGene Clipper 2 Dye Automated DNA Sequencer (Visible Genetics, Toronto, Ontario, Canada). Sequencing in the 5' and 3' directions was carried out simultaneously by using a two-dye system (Cy 5 and Cy 5.5; two-dye filter subsystem) to confirm mutations. The primers used to generate amplicons for sequencing were provided by Visible Genetics.

### W-Strain Family Genotype

The principal genetic group was determined for each isolate as described (21). In addition, specific IS*6110* insertion site mapping probes were used to determine the presence of insertions in the origin of replication and the NTF chromosomal region (20,25). The region flanking the deletion in the DR locus commonly associated with the W family was analyzed by PCR amplification and comparative hybridization (23).

### Results

### The W Family Strain Collection

The W14 fingerprint pattern was identified in a database of >13,000 clinical specimens representing approximately 9,000 patients and 3,563 distinct IS*6110* fingerprint patterns genotyped at the PHRI TB Center. From the total IS*6110* fingerprint patterns archived (N=3,563), 357 similar yet distinct patterns representing 1,498 isolates were grouped into the W family database by using standard matching criteria. Twenty isolates had an identical hybridization pattern (W14); 6 other isolates shared closely related IS*6110* fingerprint patterns (3 W23 and 3 W26). These 26 isolates with three IS*6110* patterns (W14, W23, and W26)

form the W14 group, a subgroup within the W phylogenetic lineage.

### Molecular Features of W14 Group Isolates

The W14 group was originally defined on the basis of IS*6110* patterns. The group includes three closely related fingerprints (W14, W23, and W26), with two characteristic common motifs (denoted by brackets in Figure 1A). Motifs A and B are unique to the W14 group. Southern blot hybridization of the *Pvu* II membrane with the 5'-IS*6110* probe confirmed that the difference in IS*6110* pattern between W23 and W26 was the product of two independent IS*6110* insertions, rather than the outcome of a single RFLP event (Figure 1B).

PGRS probing showed a distinct hybridization pattern (P00026) for all 26 isolates in this group (Figure 1C). The P00026 pattern was used to segregate the W14 group isolates from all other clinical samples, including other W family groups in a PGRS database of >600 isolates.

Results from five chromosomal loci (ETR-A to ETR-E) were combined as a VNTR allele profile. All isolates in the W14 group had the VNTR allele profile 42445. This allele profile was unique to the W14 group when compared with the isolates from the PHRI TB Center VNTR database (n=>500) and an independent collection archived in Durham VA Medical Center database (n=745). Together, these databases include 85 isolates of the W family, including isolates from Asia, Africa, the former Soviet Union, and the United States. However, the allele profile 42445 was closely related to other patterns found in members of the W family. The most common profile for the W family is 42435; all profiles identified to date within the W family differed from this profile by a single allele. These VNTR profiles include 32435, 42436, 42437, 4253, and the W14 group, which is defined by 42445 (19). Unlike other members of the W phylogenetic lineage, the W14 group has a unique spoligotype arbitrarily designated as S00069 (Figure 2). Spoligopattern S00069 differed from S00034 associated with other reported W family members by the absence of spacer number 40. Patterns obtained by rehybridizing the same membranes used for IS*6110* and PGRS fingerprinting with the DRab probe suggested that the disruption of spacer 40 was not due to an IS*6110* insertion (Figure 3). An IS*6110* insertion within spacer 40 would have generated two DR hybridizing bands, including one of a higher molecular weight, as found in strain CDC1551 (Figure 3) (38). Pattern S00069 was unique to the W14 group when compared with the Wadsworth spoligotype database of >2,500 isolates. The disruption of spacer 40 did not affect either chromosomal flanking region. Hence, in agreement with the S00034 spoligotype that groups all W family isolates analyzed, the region upstream from spacer 35 was deleted in isolates with S00069.

All members of the W14 group have high-level resistance (MIC>500 µg/mL) to streptomycin ($STR^R$). In addition, one W14 and one W23 isolate were resistant to INH ($INH^R$), one W14 was $INH^R$ and ethambutol ($EMB^R$) resistant, two W14 isolates were ethionamide ($ETH^R$) resistant, and one W14 was $ETH^R$ and pyrazinamide ($PZA^R$) resistant (Figure 4). Except for the 26 isolates in the W14 group, no W family isolate from a U.S. case in our collection was streptomycin monoresistant.

DNA sequencing of the gene commonly associated with streptomycin resistance, the *rpsL* gene, identified a single mutation (codon 43: AAG→AGG; Lys→Arg) in all the 26 W14 group isolates in this study. Three isolates were resis-

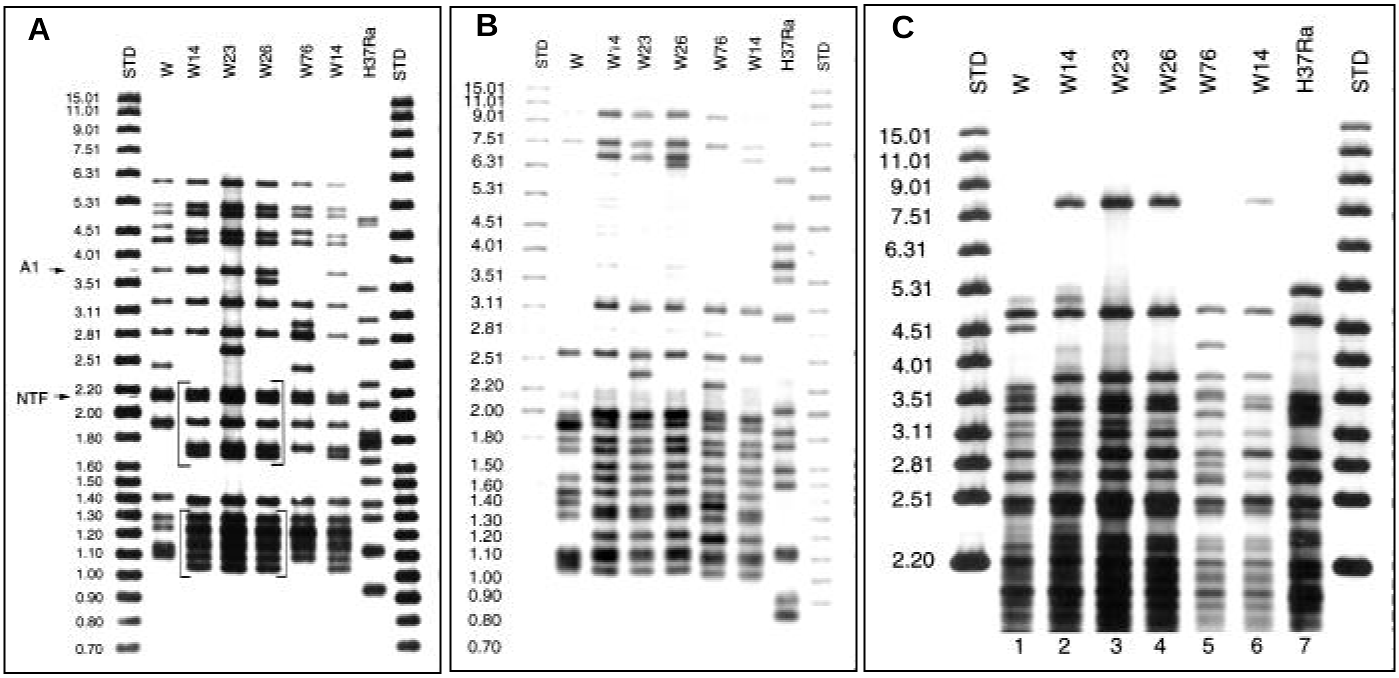

Figure 1. Southern blot hybridization of *Mycobacterium tuberculosis* isolates. A) IS*6110*-3' was used as a hybridization probe. The bracketed pattern motives regions are characteristic of the W14 family. A1 and NTF denote the bands corresponding to the IS*6110* insertions in the *dnaA-dnaN* region and the NTF locus, respectively. Lanes 1 and 9 are standard markers; lane 2: W-MDR from New York City (W index strain); lane 3, 4, 5 and 7: members of the W14 family; lane 6: W76 and lane 8: laboratory control strain H37Ra. B) Southern blot hybridization with IS*6110*-5' probe. W23 and W26 each have one additional band from W14, when hybridized with either the 3' or 5' IS*6110* probe. C) The polymorphic GC-rich repetitive sequence was used as a probe. The W14 group (W14; W23; W26) has a distinctive pattern when compared to all other isolates typed by polymorphic GC-rich repetitive sequence probe.

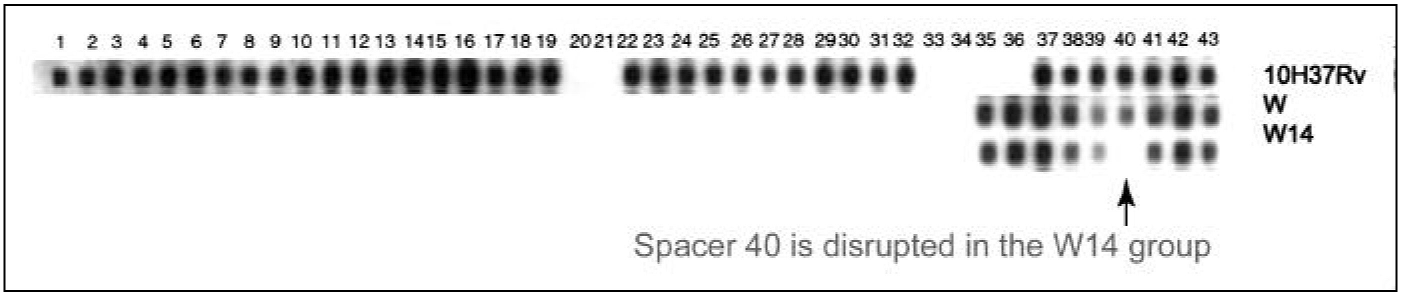

Figure 2. Spacer oligonucleotide typing (spoligotyping) of *Mycobacterium tuberculosis*. Positive hybridization with the 43 different spacer probes is denoted by black squares. W14 group has a distinctive disruption in spacer 40. Row 1: H37Ra, spoligotype S00001; 2: W-MDR, New York City isolate, spoligotype S00034; and 3: W14, spoligotype S000069.

tant to INH. Sequence analysis of the *katG* gene in these isolates revealed that the one INH$^{R}$-W23 isolate had a genetic alteration at codon 315 (AGC→ACC; Ser→Thr), while the two INH$^{R}$-W14 isolates had the same single nucleotide insertion generating a frameshift mutation at codon 283 (CTG→*A*TG), which results in a termination upstream at codon 310, previously unreported (Figure 4).

All 26 isolates in the W14 group were linked to the W family based on all the secondary typing methods used. All had *kat*G codon 463 sequence (CTG; Leu) and *gyrA* codon 95 sequence (ACC; Thr), placing them in genetic group 1 in the broad evolutionary framework outlined by Sreevatsan et al. (20,21). IS*6110* insertion site mapping showed that all 26 isolates had the A1 insertion in the origin of replication and a single IS*6110* copy in the NTF chromosomal locus (20,25).

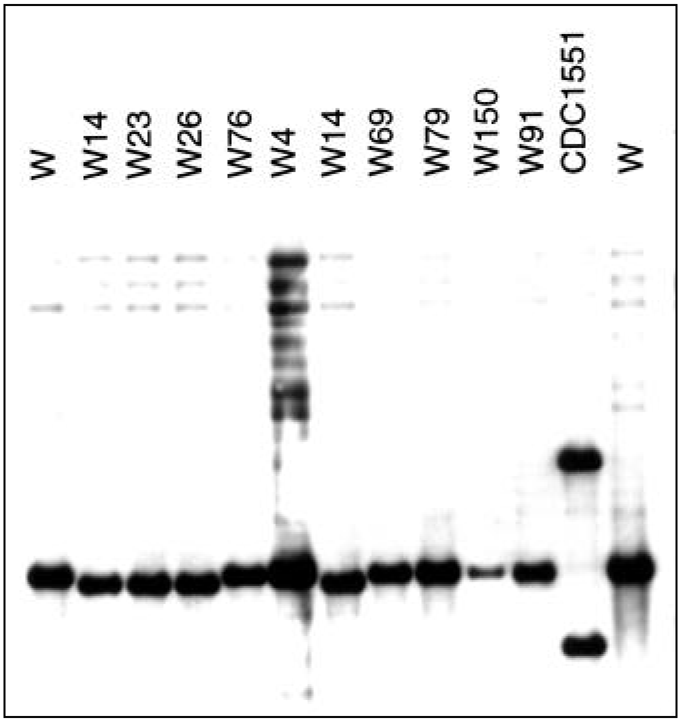

Figure 3. Southern blot hybridization with the DRab probe. The DRab probe was used to hybridize against the *Pvu*II-digested chromosomal DNA previously used for IS*6110* fingerprinting. Members of the W14 group had a single hybridizing band of a slight lower molecular weight than that of other W variants. In contrast, strain CDC1551, which is known to have an insertion within the direct-repeat locus, yielded two hybridizing bands, including one of higher molecular weight. Both hybridizing bands in strain CDC1551 can be superimposed with the IS*6110* patterns of this isolate.

### Demographic Features of the W14 Cases

For the 22 patients in the W14 group, the mean age was 40 (range: 1 to 83 yrs) (Table). All were US-born, and 17 (77%) were of non-Hispanic black origin. In patients for whom HIV serology data were available, 74% (14 of 19) were HIV seropositive. Including HIV serology, 84% (16 of 19) had at least one of the known risk factors for TB (i.e., HIV, history of incarceration, homelessness, alcohol abuse, or intravenous drug use). A review of all drug-treatment regimen records of the W14 cases showed that only one patient had streptomycin as part of TB chemotherapy.

Investigation of 18 cases from NYC identified 107 contacts (mean of 6 contacts per case). Among contacts, 24% were PPD (purified protein derivative) positive. Investigations conducted by the NYC TB Control Program did not link any patients in this cohort or identify any new contacts with active disease.

### Discussion

In this study the combination of multiple and independent molecular typing techniques permitted the identification and sub-grouping of a distinct branch of the W phylogenetic lineage, the W14 group. Twenty isolates with an identical IS*6110* pattern (W14) and an additional six (three W23 and three W26) isolates with closely related IS*6110* fingerprint patterns were identified in a fingerprint archive of >13,000 isolates. The W14 group was first typed to the "W" family fingerprint database of 1,498 isolates and then later subgrouped as a distinct branch of the W family. Five independent molecular techniques were used to confirm the distinctiveness of W14 isolates. The IS*6110* fingerprint pattern had two distinct motifs, unique within the PHRI database (Figure 1A). In addition, VNTR, PGRS, and spoligotyping patterns distinguished the W14 group from all other isolates analyzed by these methods in our collection, which includes >1,000 VNTR results, 600 PGRS images, and >2,500 spoligotype samples (Figure 1-3).

All isolates in the W14 group were resistant to high levels of streptomycin (>500 µg/mL), 20 were mono-streptomycin resistant, and the remaining 6 isolates were poly-resistant. Sequencing analysis confirmed that all isolates shared the same mutation in the *rps*L gene in codon 43, a mutation previously associated with high levels of streptomycin resistance (MIC>500; (39). The genetic alteration that directs the change of lysine to threonine in codon 43 of the *rps*L gene is one of the two most frequently reported mutations associated with streptomycin resistance: Together, they account for approximately 53% of streptomycin-resistant

Table. Demographic characteristics of patients in W14 group[a]

| Characteristics | N=22 (%) |
|---|---|
| Gender | |
| Male | 13 (59) |
| Female | 9 (49) |
| Race | |
| Non-Hispanic black | 17 (77) |
| Hispanic white | 4 (18) |
| Asian | 1 (5) |
| Age at diagnosis | |
| Median age (range) | 38 (1-83) |
| 25-50 years | 16 (73) |
| HIV serology | |
| Seropositive | 14 (64) |
| Seronegative | 5 (23) |
| Unknown | 3 (13) |
| Country of birth | |
| US born | 22 (100) |
| Foreign born | 0 (0) |
| Risk factors | |
| IVDU[b] | 7 (32) |
| Alcoholic | 9 (41) |
| Incarceration | 2 (9) |
| Homelessness | 2 (9) |
| Year of case report | |
| 1992 | 2 (9) |
| 1993 | 7 (32) |
| 1994 | 4 (18) |
| 1995 | 1 (4) |
| 1996 | 3 (14) |
| 1997 | 3 (14) |
| 1998 | 0 (0) |
| 1999 | 2 (9) |

[a]All data are presented as number (percentage) except median age.
[b]IVDU = intravenous drug use.

cases (40). Hence, this observation alone would not be sufficient to determine clonal relatedness. However, in combination with the molecular grouping, the data strongly suggest that these strains are clonal and are the progeny of a single streptomycin-resistant predecessor.

Treatment records showed that only one patient received two months of streptomycin in combination with INH and rifampin. This regimen was changed when susceptibility data were available. The absence of streptomycin exposure in the patients with the W14 group of isolates further supports the thesis that each of these patients was infected with a streptomycin-resistant isolate rather than acquiring resistance while on therapy.

There were 295 streptomycin mono-resistant isolates from 295 patients reported in NYC during 1993 and 1995 to 1999; 49% of these were from foreign-born patients. Of the 150 U.S.-born patients with mono-streptomycin resistant TB reported in NYC during the same period, 83 isolates were genotyped (55%); 31% (n=26) belonged to the W14 group. Genotyping data of 187 mono-streptomycin-resistant clinical isolates, including the 83 from NYC, identified the W14 group as the only epidemiologically important cluster of patients. Six additional, unrelated clusters of two cases each were identified in the PHRI fingerprint database. From the PHRI archive, no streptomycin-susceptible isolates with the W14 molecular characteristics have been identified to date from NYC or other locations (samples fingerprinted: n= >12,000; NYC: n= >6,000). Taken together, the molecular data point to the acquisition of streptomycin resistance before dissemination of this strain into the community.

Several strains have continued to acquire additional secondary drug resistances (Figure 4), including INH, ETH, PZA, and EMB (W14: 1 $STR^R$ $INH^R$, 1 $STR^R$ $INH^R$ $EMB^R$, 2 $STR^R$ $ETH^R$, 1 $STR^R$ $PZA^R$ $ETH^R$; W23: 1 $STR^R$ $INH^R$). None of the isolates examined acquired resistance to rifampin. In a previous study, we elucidated a plausible pathway for the evolution of the W-MDR strain in NYC (W-index strain) in the early 1990s (17). In that study, the emergence and spread of the W-MDR strain in NYC was believed to have started by acquisition of streptomycin resistance, followed by INH and rifampin resistance, but the outbreak did not occur until the MDR-genotype was developed. In contrast, the W14 group of isolates spread after streptomycin resistance was acquired; subsequently, additional resistance developed, creating a group of poly-resistant variants. Sequencing data in combination with IS*6110* Southern blot hybridization were used to demonstrate that INH resistance had developed on two occasions independently, once in W14 and once in a W23 (Figure 4) isolate. The W23 *katG* substitution on codon 315 is found on the same codon as in the W-MDR index strain but involves a different nucleotide. DNA sequence analysis of different resistance target genes provides molecular markers to speculate the stepwise building of polyresistant strains.

Since the late 1980s, molecular methods have been gradually integrated into the study of TB epidemiology and control. In addition to augmenting conventional epidemiologic investigations, molecular typing has been used to identify previously unrecognized point-source cases and in two reports was used to confirm transmission in a social setting (41,42). More recently, molecular typing with surveillance data uncovered an epidemiologically significant strain cluster (n=43), the W4 group, from New Jersey, that has no apparent common point source or patient links (19). The concordance of demographic and geographic data with molecular methods and the lack of concrete links in the W4 cases suggest a combination of reactivation and recent transmission. Likewise in this study, despite the demographic, geographic and molecular grouping, we could not establish any patient linkage in the W14 group. Nonetheless, the extent and validity of the molecular data demonstrated that these 22 patients were infected with either the same strain (16 W14 cases) or one of two closely related isolates (3 W23 or 3 W26).

This study has a number of limitations. PHRI's collection from NYC reflects a convenience sample that is approximately 44% of the total number of culture-positive TB cases reported to the NYC TB Control Program in 1992 to 1999. Of the W14 group patients, all were U.S.-born, >70% were HIV positive, and most (73%) were 25 to 50 years of age at diagnosis. Despite the demographic homogeneity in these

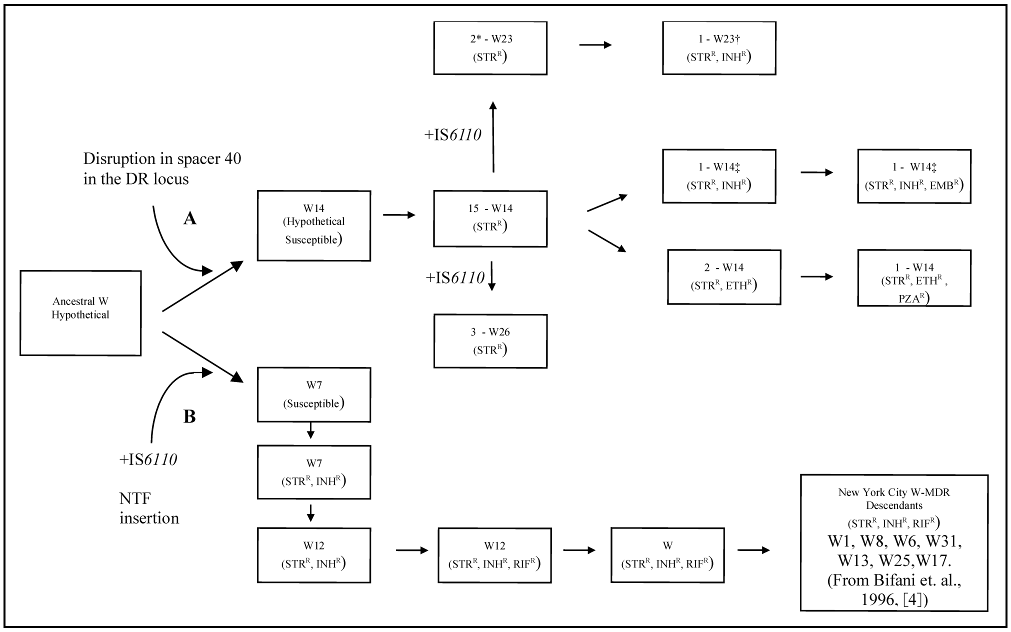

Figure 4. Schematic diagram on a plausible evolutionary pathway of the W14 *Mycobacterium tuberculosis* group, compared with the New York City W-MDR (4). All members of the W14 group lineage have single mutation on codon 43 of the *rps*L target gene associated with high levels of streptomycin resistance (STR$^R$). Note that isoniazid-resistant (INH$^R$) W23 and W14 branches have a distinct *katG* mutation. The W23 *katG* substitution on codon 315 is found on the same codon as in the W-MDR index strain but involves a different nucleotide; the W14 *katG* insertion on codon 283 has not been previously reported. ETH$^R$ = ethionamide resistant; PZA$^R$ = pyrazinamide resistant; EMB = ethambutol resistant; R= resistant.

patients, no epidemiologic links were established. Furthermore, the limited sampling suggests an actual denominator of W14 group cases larger than reported in this study, which aids in explaining the inability to establish epidemiologic links in the patients. Patient interviews were initially conducted as part of routine contact investigation; patients were not reinterviewed once molecular cluster information was available. Therefore, in-depth investigation of cases that appeared from molecular and surveillance data to be related was not available, which limits the inferences we can make on the observed lack of epidemiologic links.

Given the lack of overt epidemiologic links indicative of recent transmission between cases, the possibility that the W14 strain group is endemic to the region should be considered. However, the streptomycin monoresistance suggests that this strain must have spread after the introduction and application of this drug for TB treatment in 1944. It is surprising that not a single susceptible, related strain has been identified in the region even following extensive molecular analysis of other W variants. If the W14 or its predecessor were endemic, only the streptomycin-resistant variant has managed to spread, again suggesting that this dissemination depended on a single event, and yet, contact investigation has failed to establish a link. We speculate that the W14 cluster with the large young HIV- seropositive population is likely the result of recent transmission; however, the lack of patient-to-patient links suggests a subgroup of cases caused by an endemic strain that developed in NYC sometime after streptomycin was introduced in 1944.

By using multiple independent molecular markers, we made inferences on the strains' recent evolution. This study highlights the importance of tracking all types of drug-resistant strains to prevent the sequential development of multi-drug-resistant strains. The utility that these methods lend to TB control will rely on the efficiency of integrating both surveillance and contact-tracing information with the molecular data in a proactive manner for more well-informed TB investigations.

### Acknowledgments

We thank H. Marasco, A. Ravikovitch, and W. Eisner for assistance with patient and fingerprint databases. We thank Visible Genetics for providing us with the Clipper Sequencer and sequencing kits on a trial basis.

This research was supported in part by CDC's National Tuberculosis Genotyping and Surveillance Network Cooperative Agreement. This is publication 75 from the Public Health Research Institute, Tuberculosis Center.

Dr. Bifani was a graduate student in the Public Health Research Institute Tuberculosis Center in New York City at the time this article was written. The center was established in January 1992 in response to the reemergence of TB in New York. He is currently at the Institut Pasteur de Lille France working as a postdoctoral fellow.

### References

1. Chaves F, Dronda F, Alonso-Sanz M, Noriega AR. Evidence of exogenous reinfection and mixed infection with more than one strain of *Mycobacterium tuberculosis* among Spanish HIV-infected inmates. AIDS 1999;13:615-20.
2. Moro ML, Gori A, Errante I, Infuso A, Franzetti F, Sodano L, et al. An outbreak of multidrug-resistant tuberculosis involving HIV-infected patients of two hospitals in Milan, Italy. Italian Multidrug-Resistant Tuberculosis Outbreak Study Group. AIDS 1998;12:1095-102.
3. Pfyffer GE, Strassle A, Rose N, Wirth R, Brandli O, Shang H. Transmission of tuberculosis in the metropolitan area of Zurich: a 3 year survey based on DNA fingerprinting [see comments]. Eur Respir J 1998;11:804-8.
4. Edlin BR, Tokars JI, Grieco MH, Crawford JT, Williams J, Sordillo EM, et al. An outbreak of multidrug-resistant tuberculosis among hospitalized patients with the acquired immunodeficiency syndrome [see comments]. N Engl J Med 1992;326:1514-21.
5. Kenyon TA, Ridzon R, Luskin-Hawk R, Schultz CV, Paul WS, Valway SE, et al. A nosocomial outbreak of multidrug-resistant tuberculosis. Ann Intern Med 1997;127:32-6.
6. Sahm DF, Tenover FC. Surveillance for the emergence and dissemination of antimicrobial resistance in bacteria. Infect Dis Clin North Am 1997;11:767-83.

7. Small PM, McClenny NB, Singh SP, Schoolnik GK, Tompkins LS, Mickelsen PA. Molecular strain typing of *Mycobacterium tuberculosis* to confirm cross-contamination in the mycobacteriology laboratory and modification of procedures to minimize occurrence of false-positive cultures. J Clin Microbiol 1993;31:1677-82.
8. Bauer J, Thomsen VO, Poulsen S, Andersen AB. False-positive results from cultures of *Mycobacterium tuberculosis* due to laboratory cross-contamination confirmed by restriction fragment length polymorphism. J Clin Microbiol 1997;35:988-91.
9. Carricajo A, Vincent V, Berthelot P, Gery P, Aubert G. Mycobacterial cross-contamination of bronchoscope detected by molecular techniques [letter]. J Hosp Infect 1999;42:252-3.
10. Yang ZH, de Haas PE, Wachmann CH, van Soolingen D, van Embden JD, Andersen AB. Molecular epidemiology of tuberculosis in Denmark in 1992. J Clin Microbiol 1995;33:2077-81.
11. Samper S, Iglesias MJ, Rabanaque MJ, Lezcano MA, Vitoria LA, Rubio MC, et al. The molecular epidemiology of tuberculosis in Zaragoza, Spain: a retrospective epidemiological study in 1993. Int J Tuberc Lung Dis 1998;2:281-7.
12. Small PM, Shafer RW, Hopewell PC, Singh SP, Murphy MJ, Desmond E, et al. Exogenous reinfection with multidrug-resistant *Mycobacterium tuberculosis* in patients with advanced HIV infection [see comments]. N Engl J Med 1993;328:1137-44.
13. van Rie A, Warren R, Richardson M, Victor RC, Gie RP, Enarson DA, et al. Exogenous reinfection as a cause of recurrent tuberculosis after curative treatment [see comments]. N Engl J Med 1999;341:1174-9.
14. Frieden TR, Sherman LF, Maw KL, Fujiwara PI, Crawford JT, Nivin B, et al. A multi-institutional outbreak of highly drug-resistant tuberculosis: epidemiology and clinical outcomes [see comments]. JAMA 1996;276:1229-35.
15. Valway SE, Greifinger RB, Papania M, Kilburn JO, Woodley C, DiFerdinando GT, et al. Multidrug-resistant tuberculosis in the New York State prison system, 1990-1991. J Infect Dis 1994;170:151-6.
16. Moss AR, Alland D, Telzak E, Hewlett D Jr, Sharp V, Chillade P, et al. A city-wide outbreak of a multiple-drug-resistant strain of *Mycobacterium tuberculosis* in New York. Int J Tuberc Lung Dis 1997;1:115-21.
17. Bifani PJ, Plikaytis BB, Kapur V, Stockbauer K, Pan X, Lutfey ML, et al. Origin and interstate spread of a New York City multidrug-resistant *Mycobacterium tuberculosis* clone family [see comments]. JAMA 1996;275:452-7.
18. Agerton TB, Valway SE, Blinkhorn RJ, Shilkret KL, Reves R, Schluter WW, et al. Spread of strain W, a highly drug-resistant strain of *Mycobacterium tuberculosis*, across the United States. Clin Infect Dis 1999;29:85-95.
19. Bifani PJ, Mathema B, Liu Z, Moghazeh SL, Shopsin B, Tempalski B, et al. Identification of a W variant outbreak of *Mycobacterium tuberculosis* via population-based molecular epidemiology. JAMA 1999;282:2321-7.
20. Kurepina NE, Sreevatsan S, Plikaytis BB, Bifani PJ, Connell ND, Donnelly RJ, et al. Characterization of the phylogenetic distribution and chromosomal insertion sites of five IS6110 elements in *Mycobacterium tuberculosis*: non-random integration in the dnaA-dnaN region. Tuber Lung Dis 1998;79:31-42.
21. Sreevatsan S, Pan X, Stockbauer KE, Connell ND, Kreiswirth BN, Whittam TS, et al. Restricted structural gene polymorphism in the *Mycobacterium tuberculosis* complex indicates evolutionarily recent global dissemination. Proc Natl Acad Sci U S A 1997;94:9869-74.
22. van Embden JD, van Gorkom T, Kremer K, Jansen R, van Der Zeijst BA, Schouls LM. Genetic variation and evolutionary origin of the direct repeat locus of *Mycobacterium tuberculosis* complex bacteria. J Bacteriol 2000;182:2393-401.
23. van Rie A, Warren RM, Beyers N, Gie RP, Classen CN, Richardson M, et al. Transmission of a multidrug-resistant *Mycobacterium tuberculosis* strain resembling "strain W" among noninstitutionalized, human immunodeficiency virus-seronegative patients. J Infect Dis 1999;180:1608-15.
24. van Soolingen D, Qian L, de Haas PE, Douglas JT, Traore H, Portaels F, et al. Predominance of a single genotype of *Mycobacterium tuberculosis* in countries of east Asia. J Clin Microbiol 1995;33:3234-8.
25. Plikaytis BB, Marden JL, Crawford JT, Woodley CL, Butler WR, Shinnick TM. Multiplex PCR assay specific for the multi-drug-resistant strain W of *Mycobacterium tuberculosis*. J Clin Microbiol 1994;32:1542-6.
26. Anh DD, Borgdorff MW, Van LN, Lan NT, van Gorkom T, Kremer K, et al. *Mycobacterium tuberculosis* Beijing genotype emerging in Vietnam. Emerg Infect Dis 2000;6:302-5.
27. Namwat W, Luangsuk P, Palittapongarnpim P. The genetic diversity of *Mycobacterium tuberculosis* strains in Thailand studied by amplification of DNA segments containing direct repetitive sequences. Int J Tuberc Lung Dis 1998;2:153-9.
28. Lutfey M, Della-Latta P, Kapur V, Palumbo LA, Gurner D, Stotzky G, et al. Independent origin of mono-rifampin-resistant *Mycobacterium* tuberculosis in patients with AIDS [see comments]. Am J Respir Crit Care Med 1996;153:837-40.
29. Friedman CR, Quinn GC, Kreiswirth BN, Perlman DC, Salomon N Schluger N, et al. Widespread dissemination of a drug-susceptible strain of *Mycobacterium tuberculosis* [see comments]. J Infect Dis 1997;176:478-84.
30. Nivin B, Fujiwara PI, Hannifin J, Kreiswirth BN. Cross-contamination with *Mycobacterium tuberculosis*: an epidemiological and laboratory investigation. Infect Control Hosp Epidemiol 1998;19:500-3.
31. Fujiwara PI, Cook SV, Rutherford CM, Crawford JT, Glickman SE, Kreiswirth BN, et al. A continuing survey of drug-resistant tuberculosis, New York City, April 1994. Arch Intern Med 1997;157:531-6.
32. van Embden JD, Cave MD, Crawford JT, Dale JW, Eisenach KD, Gicquel B, et al. Strain identification of *Mycobacterium tuberculosis* by DNA fingerprinting: recommendations for a standardized methodology [see comments]. J Clin Microbiol 1993;31:406-9.
33. Chaves F, Yang Z, el Hajj H, Alonso M, Burman WJ, Eisenach KD, et al. Usefulness of the secondary probe pTBN12 in DNA fingerprinting of *Mycobacterium tuberculosis*. J Clin Microbiol 1996;34:1118-23.
34. Kamerbeek J, Schouls L, Kolk A, van Agterveld M, van Soolingen D, Kuijper S, et al. Simultaneous detection and strain differentiation of *Mycobacterium tuberculosis* for diagnosis and epidemiology. J Clin Microbiol 1997;35:907-14.
35. Frothingham R, Meeker-O'Connell WA. Genetic diversity in the *Mycobacterium tuberculosis* complex based on variable numbers of tandem DNA repeats. Microbiology 1998;144:1189-96.
36. Standards National Committee for Clinical Laboratory Standards. Antimycobacterial susceptibility testing. Proposed standard M24-P. Villanova (PA): The Committee;1990.
37. Miller MA, Thibert L, Desjardins F, Siddiqi SH, Dascal A. Testing of susceptibility of *Mycobacterium tuberculosis* to pyrazinamide: comparison of Bactec method with pyrazinamidase assay. J Clin Microbiol 1995;33:2468-70.
38. Plikaytis BB, Kurepina N, Woodley CL, Fleischmann R, Kreiswirth B, Shinnick TM. Multiplex PCR assay to aid in the identification of the highly transmissible *Mycobacterium tuberculosis* strain CDC1551. Tuber Lung Dis 1999;79:273-8.
39. Cooksey RC, Morlock GP, McQueen A, Glickman SE, Crawford JT. Characterization of streptomycin resistance mechanisms among *Mycobacterium tuberculosis* isolates from patients in New York City. Antimicrob Agents Chemother 1996;40:1186-8.
40. Ramaswamy S, Musser JM. Molecular genetic basis of antimicrobial agent resistance in *Mycobacterium tuberculosis:* 1998 update. Tuber Lung Dis 1998;79:3-29.
41. Sterling TR, Thompson D, Stanley RL, McElroy PD, Madison A, Moore K, et al. A multi-state outbreak of tuberculosis among members of a highly mobile social network: implications for tuberculosis elimination. Int J Tuberc Lung Dis 2000;4:1066-73.
42. Yaganehdoost A, Graviss EA, Ross M, Adams GJ, Ramaswamy S, Wanger A, et al. Complex transmission dynamics of clonally related virulent *Mycobacterium tuberculosis* among predominantly HIV-positive gay males associated with "barhopping" supports location-based control strategies. J Infect Dis 1999;180:1245-51.
